# Adding Guar Gum to High‐Fat Diets Ameliorates Fish Growth, Gut Histology, Gut Microbiota Composition, and Intestinal Inflammation in Common Carp

**DOI:** 10.1155/anu/2722361

**Published:** 2026-01-16

**Authors:** Weijun Chen, Shiyang Gao, Xiaoyu Zhao, Na Zhao, Ping Sun, Lei Han

**Affiliations:** ^1^ College of Animal Science and Technology, Henan University of Science and Technology, Luoyang, Henan, China, haust.edu.cn; ^2^ College of Agricultural Equipment Engineering, Henan University of Science and Technology, Luoyang, Henan, China, haust.edu.cn

**Keywords:** common carp, guar gum, gut health, intestinal microbiota, oxidative stress

## Abstract

The purpose of this research was to investigate how adding dietary guar gum to high‐lipid diets affected the fish growth and gut health of common carp (*Cyprinus carpio*). A normal‐lipid diet (5% crude lipid; control) and four high‐lipid diets (10% crude lipid) with 0% (high‐fat [HF]), 0.3% (GG0.3), 1% (GG1), and 3% (GG3) of guar gum were developed and fed to fish (4.53 g) for 8 weeks. The findings showed that HF induced impairment of intestinal morphology and mucosal barrier, oxidative stress, gut dysbiosis, and gut inflammation. Compared to the HF, guar gum‐containing diets substantially improved gut villus height, upregulated the expression levels of *nuclear factor erythroid 2-related factor 2* and *zonula occludens-1*, and downregulated the expression levels of *toll-like receptor 1* (*tlr1*), *tlr5*, *myeloid differentiation factor 88*, *interleukin-1β* (*il-1β*), *il-6*, and *il-8*. Moreover, the GG0.3 and GG1 diets dramatically increased *catalase (cat)* and *occludin* expression levels. Furthermore, the GG1 and GG3 diets improved the microbiota composition by increasing Fusobacteria and *Cetobacterium* abundance while lowering Proteobacteria, *Acidovorax*, *Acinetobacter*, *Serratia*, and *Comamonas* abundance. Correlation analysis revealed that guar gum improved gut health by modulating gut microbiota and tight junction proteins. The findings indicated that guar gum can ameliorate HF diet‐induced intestinal damage in fish.

## 1. Introduction

China dominates global common carp aquaculture production, contributing 67.72% of the 4.18 million tons output [1]. High‐fat (HF) diets are usually used to reduce production costs in common carp farming because of the protein‐sparing effect of lipids [2]. While HF diets lower production costs in common carp aquaculture, they also lead to gut inflammation [3, 4]. This seriously impedes the sustainable development of common carp farming.

HF diets induce gut inflammation in fish primarily through gut dysbiosis and intestinal barrier compromise [5, 6]. These diets disrupt gut microbiota composition [7, 8], increasing the production of toxins (e.g., lipopolysaccharides [LPS]) and susceptibility to gut inflammation [9]. Moreover, HF diets impair the gut barrier by downregulating tight junction proteins, exposing lamina propria immune cells to luminal antigens, and promoting enteritis [10, 11]. Therefore, strategies for improving gut dysbiosis and enhancing tight junction protein expression can be of great potential in solving problems of farmed common carp fed HF diets.

Guar gum is a naturally occurring galactomannan polysaccharide derived from the guar plant (*Cyamopsis tetragonoloba*) seeds. Characterized by a linear chain of mannose units with galactose side groups, guar gum exhibits unique structural properties that contribute to its high solubility and viscosity in aqueous solutions [12]. These properties enable guar gum to function effectively as a thickening agent, stabilizer, and emulsifier in various food and pharmaceutical applications [13]. Moreover, as a soluble dietary fiber, guar gum plays a significant role in modulating gut health [14]. Recent studies have highlighted the prebiotic potential of guar gum, demonstrating its capacity to improve gut microbiota composition and upregulate tight junction proteins. Previous studies have shown that guar gum can alleviate intestinal dysbiosis and stimulate the production of short‐chain fatty acids (SCFAs) in mammals [15, 16]. SCFAs can boost the function of the gut barrier and reduce inflammation [17]. Moreover, an investigation on mice has revealed that guar gum upregulated tight junction proteins’ expression, such as *zonula occludens-1* (*zo-1*) [18].

While guar gum is recognized for its potential benefits, the literature lacks clarity on its specific impacts on gut health in aquatic species—particularly when administered under HF diets that induce gut damage. This study systematically investigates the protective effects of dietary guar gum on the intestine of common carp (*Cyprinus carpio*) fed a HF diet, aiming to elucidate the protective mechanisms of guar gum against HF diet–induced enteritis.

## 2. Materials and Methods

### 2.1. Ethical Statement

The study’s experimental design and procedures were approved by the Animal Protection and Utilization Committee of the Henan University of Science and Technology. All animal‐related research activities were conducted in strict accordance with the guidelines for the care and use of laboratory animals.

### 2.2. Guar Gum and Test Diets

Guar gum powder was white to off‐white and odorless, with 88.5% galactomannan, 1.1% ash, and 5.9% protein (see the list in Table S1). The galactomannan molecule consisted of chains of (1–4)‐linked *β*‐d‐mannopyranosyl units and single *α*‐d‐galactopyranosyl units joined by (1–6) links. The primary chain of galactomannan was composed of d‐mannose, whereas the side chain was composed of d‐galactose. The ratio of mannose to galactose in guar gum is 1.6:1. The viscosity was measured at 5200 mPa·s after being dissolved in 1% aqueous solution for 24 h at 25°C (Table S1).

Five experimental diets were formulated with differing lipid levels. The normal‐fat control diet (control) contained 5.8% crude lipid, while the HF diet was formulated at 10.8%. Three additional isolipidic diets incorporated guar gum at concentrations of 0.3% (GG0.3), 1% (GG1), and 3% (GG3) into HF. The composition of control and HF is shown in Table 1. After pulverizing and passing through a 200 μm screen, all materials were properly mixed using a mixer and conditioned with ~25% distilled water. The pellets (2 mm) were made, dried, and stored at 4°C.

**Table 1:** Proximate composition of basal diets (% dry matter).

Ingredients	Control	HF
Casein	28.0	28.0
Gelatin	7.0	7.0
Dextrin	25.0	25.0
Soybean oil	5.0	10.0
Mineral and vitamin premix	1.0	1.0
Vitamin C	1.0	1.0
Ca(H_2_PO_4_)_2_	2.5	2.5
Choline chloride	0.5	0.5
Guar gum	0.0	0.0
Cellulose	30.0	25.0
Proximate composition (% dry matter)		
Moisture	9.97	9.83
Crude protein	31.43	31.63
Crude lipid	5.80	10.84
Ash	2.48	2.60

### 2.3. Experimental Fish and Rearing Conditions

Juvenile common carp were procured from a Guangzhou fish farm and laboratory‐acclimated for 2 weeks. Following a 24‐h starvation period, experimental fish (4.53 ± 0.05 g) were assigned at random to 15 tanks (200 L tanks; 30 fish/tank). During 8 weeks, fish received two meals per day to satiation. Prior to each feeding, the water in the tank was changed and feces were removed. Rearing conditions comprised a 12:12 h light: dark cycle, water temperature of 25.5 ± 0.6°C, dissolved oxygen≥ 6 mg/L, and ammonia ≤ 0.1 mg/L.

### 2.4. Sample Collection

Six hours post‐feeding, three fish per tank were sacrificed by lethal anesthesia using benzocaine (100 mg/L). Then the intestinal digesta was gathered in a sterilized tube and stored at −80°C for the analysis of intestinal microbiota.

Twenty‐four hours following the last meal, common carp in every tank were put under anesthesia and weighed for the assessment of fish growth. From each of the three replicate tanks per treatment, nine fish were sampled, with three specimens allocated to each analytical procedure: Bouin’s solution‐preserved distal intestines for histology, plasma collection for antioxidant status assessment, and gut tissue acquisition for gene expression profiling. Intestine and plasma samples for investigation of antioxidant indicators or gene expression were kept at −80°C.

### 2.5. Analysis of Diet Composition

The conventional protocols were employed to ascertain diet composition [19]. Moisture content was determined by drying at 105°C to constant mass. Crude protein was calculated by multiplying the nitrogen content of the samples by 6.25. Crude lipid in feed was extracted using petroleum ether. Ash was quantified by placing samples into porcelain crucibles and subjecting them to a furnace (600°C) for 2 h.

### 2.6. Determination of Plasma Antioxidant‐Related Indicators

According to Nanjing Jiancheng kit protocols, the activity levels of total superoxide dismutase (T‐SOD), catalase (CAT), and glutathione peroxidase (GPx), and the contents of reduced glutathione (GSH), malonaldehyde (MDA), protein carbonyls (PC), and total protein were measured via Tecan’s Infinite 200 PRO microplate reader.

### 2.7. Gut Histology

Fixed gut samples were dehydrated using graded concentrations of ethanol (30%–70%), embedded in paraffin, and sectioned transversely (4 μm thickness). Then, hematoxylin and eosin were used to stain the sections. Villus length and width, muscular thickness, and perimeter ratio (PR) were determined by examining 10 different visual fields for each sample.

### 2.8. Analysis of Gut Microbiota

Intestinal microbiota profiling was performed according to established protocols [20]. Total DNA underwent extraction and quantification. The V3‐V4 hypervariable region of the 16S rRNA gene was amplified with primers B341F (5′‐CCTACGGGNGGCWGCAG‐3′) and B785R (5′‐GACTACHVGGGTATCTAATCC‐3′). PCR reactions (25 μL) contained 2.5 μL DNA template, 12.5 μL 2× KAPA Hifi HotStart ReadyMix, 0.25 μL each primer (25 μmol/L), and 9.5 μL PCR‐grade water. Thermal cycling conditions were 95°C for 3 min; 25 cycles of 95°C (30 s), 55°C (30 s), 72°C (30 s) and final extension at 72°C for 5 min.

Amplified products were electrophoresed, purified, quantified, and subjected to a second PCR. Amplicon sequencing was conducted on an Illumina MiSeq platform. Raw reads were filtered to remove primer sequences, merged into high‐quality contigs, and screened for chimeras to produce effective sequences.

These sequences were clustered into operational taxonomic units (OTUs) at 97% sequence similarity using Vsearch (v2.3.4). OTUs were taxonomically classified and annotated against the SILVA database. Species abundance tables across taxonomic ranks were generated with QIIME (v1.7.0). Alpha diversity indices (Chao1, ACE, Shannon, and Simpson) were calculated from OTU data. Beta diversity was evaluated via principal coordinate analysis (PCoA) and UPGMA clustering. Differential abundance analysis between groups was performed using STAMP software.

### 2.9. Quantitative Real‐Time PCR (qRT‐PCR)

Our previous methods [21] were employed to conduct the total RNA extraction, reverse transcription, and mRNA quantification. All chosen gene sequences were derived from the published work on common carp (Table S2). qRT‐PCR was performed on a LightCycler 480 II system (Roche Diagnostics) using SYBR Green supermix (Bio‐Rad, USA). *β*‐actin served as the endogenous reference gene for template normalization. Gene expression levels in experimental dietary groups were calculated relative to the control group.

### 2.10. Calculations and Statistics

The growth‐related parameters and PR were determined using the methods below:

Specific growth rate (SGR) (%/d) = 100 × (ln [final body weight] −ln [initial body weight])/days

Fish survival (%) = 100 × final fish number/initial fish number

PR = IP/EP

Where IP was the internal perimeter of the intestine lumen, and EP was the external perimeter of the intestine.

All data were presented in the current study as arithmetic means with associated standard deviation. Before doing the analysis, all the data were subjected to a one‐way ANOVA and then the Duncan multiple range test. The significance threshold for differences was set at *p*  < 0.05.

## 3. Results

### 3.1. Common Carp Growth

Fish in the GG0.3 group exhibited a higher FBW and SGR than those given the HF diet (*p* < 0.05). Addition of guar gum did not affect fish survival (Figure 1).

Figure 1Fish growth and survival of common carp. (A) Final body weight. (B) SGR, specific growth rate. (C) Fish survival. Bars with different lowercase letters exhibit significant differences (*p* < 0.05).

**Figure:**
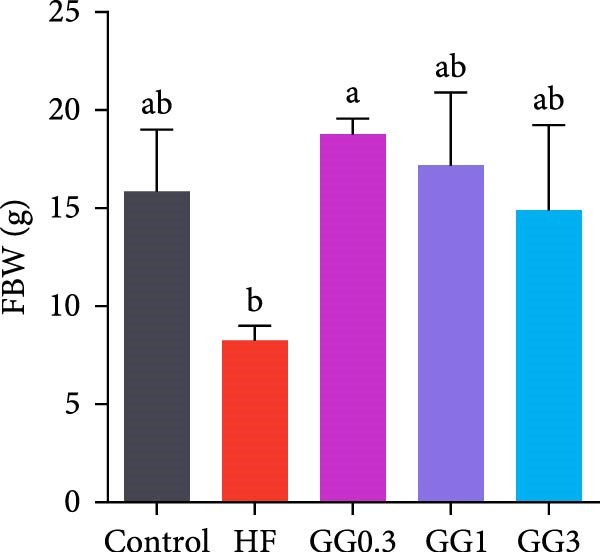
(A)

**Figure:**
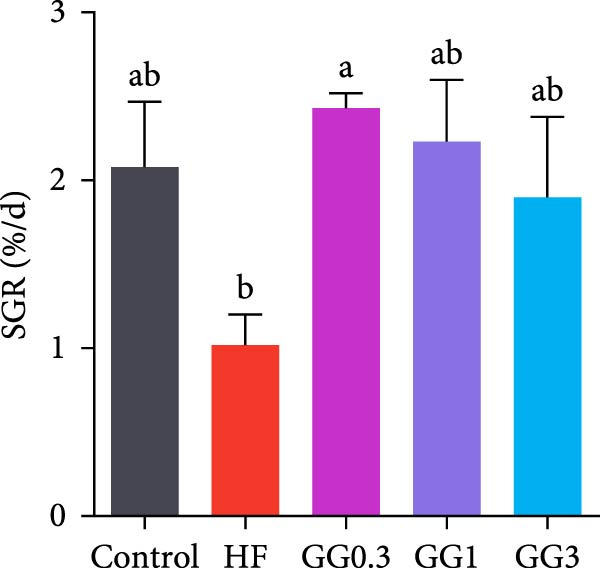
(B)

**Figure:**
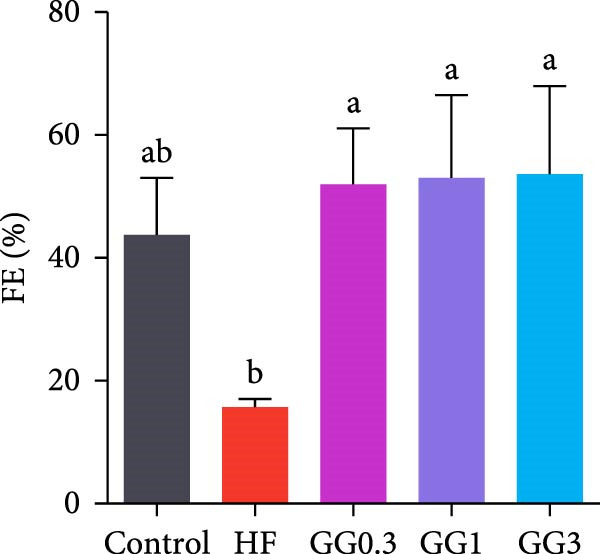
(C)

### 3.2. Intestinal Microbiota Composition

Following quality control, 3231 OTUs were identified with 97% sequence similarity. The number of OTUs among groups ranged from 142 to 313 (Table 2). In contrast to the control, the HF and GG0.3 diets induce a significant increase in the Shannon index and a fall in the Simpson index. However, fish given diets GG1 and GG3 demonstrated a greater Simpson index and a lower Shannon index, as opposed to those given the diet HF (*p* < 0.05).

**Table 2:** Alpha diversity of the microbial sequencing of common carp intestine.

Groups	99% similarity				
	OTUs	Chao	ACE	Shannon	Simpson
Control	203.00 ± 11.57	231.13 ± 19.79	242.07 ± 19.06	1.21 ± 0.33^b^	0.58 ± 0.10^a^
HF	313.50 ± 90.93	326.74 ± 88.75	344.88 ± 79.68	2.99 ± 0.69^a^	0.17 ± 0.08^b^
GG0.3	227.00 ± 16.74	249.36 ± 21.41	251.12 ± 20.98	2.59 ± 0.10^a^	0.15 ± 0.03^b^
GG1	191.33 ± 49.75	281.18 ± 92.09	279.65 ± 90.37	0.95 ± 0.11^b^	0.64 ± 0.08^a^
GG3	142.50 ± 15.84	168.20 ± 21.41	180.17 ± 22.53	1.22 ± 0.12^b^	0.49 ± 0.06^a^

*Note:* Data in the same column with distinct superscript letters exhibit statistical differences (*p* < 0.05).

To investigate the variety of intestinal microbial composition among all groups, a UPGMA clustering tree and PCoA were employed (Figure S1). The clustering of intestinal microbial communities in GG0.3 and HF samples displayed obvious separation compared with the control, GG1, and GG3 (Figure S1A). Likewise, PCoA demonstrated that samples in the control, GG1, and GG3 groups were closely clustered and clearly separated from the GG0.3 and HF groups (Figure S1B).

Fusobacteria, Proteobacteria, Bacteroidetes, Firmicutes, Planctomycetes, Verrucomicrobia, Actinobacteria, and Chlamydiae dominated the microbiota community structure at the phylum level (Figure 2A). Fusobacteria abundance in fish fed HF was markedly reduced (*p* < 0.05) compared to the control, whereas Proteobacteria abundance increased concurrently (*p* < 0.05). Comparing the GG1 and GG3 diets to the HF diet, there was a significant drop in Proteobacteria abundance and an increase in Fusobacteria abundance (*p* < 0.05). Fish in the GG0.3 group exhibited a greater Firmicutes abundance compared to GG1 or control, but a lower Fusobacteria abundance than GG1 or GG3 group (Figure 2B).

Figure 2Intestinal microbiota composition in fish fed experimental diets. (A) Dominant microbial phyla. (B) Microbial phyla with significant differences. (C) Dominant microbial genera. (D) Microbial genera with significant differences.

**Figure:**
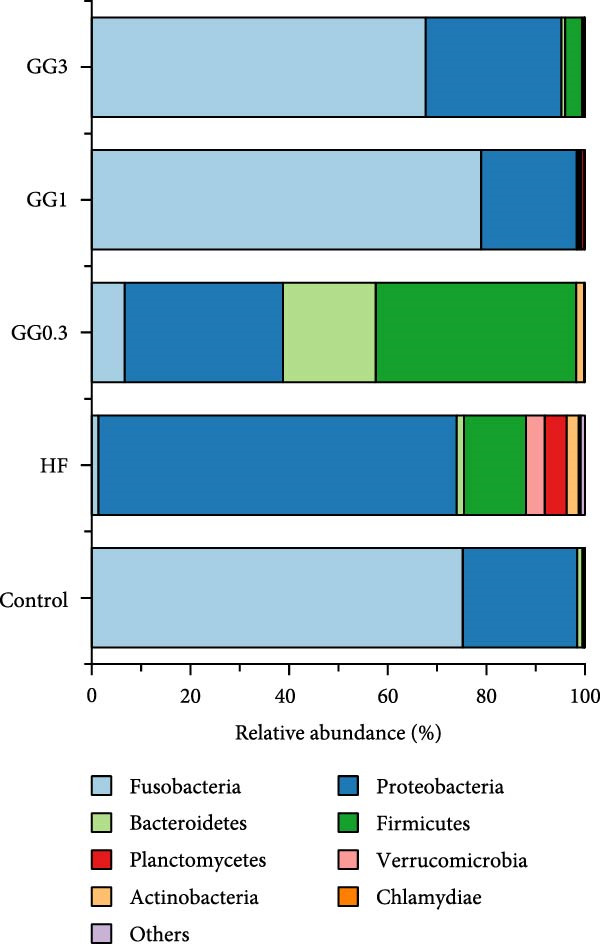
(A)

**Figure:**
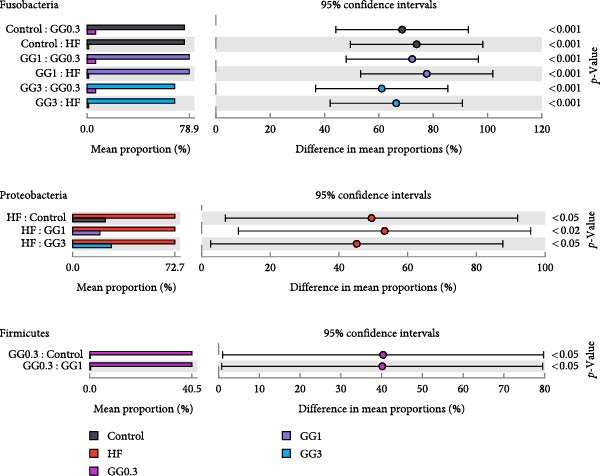
(B)

**Figure:**
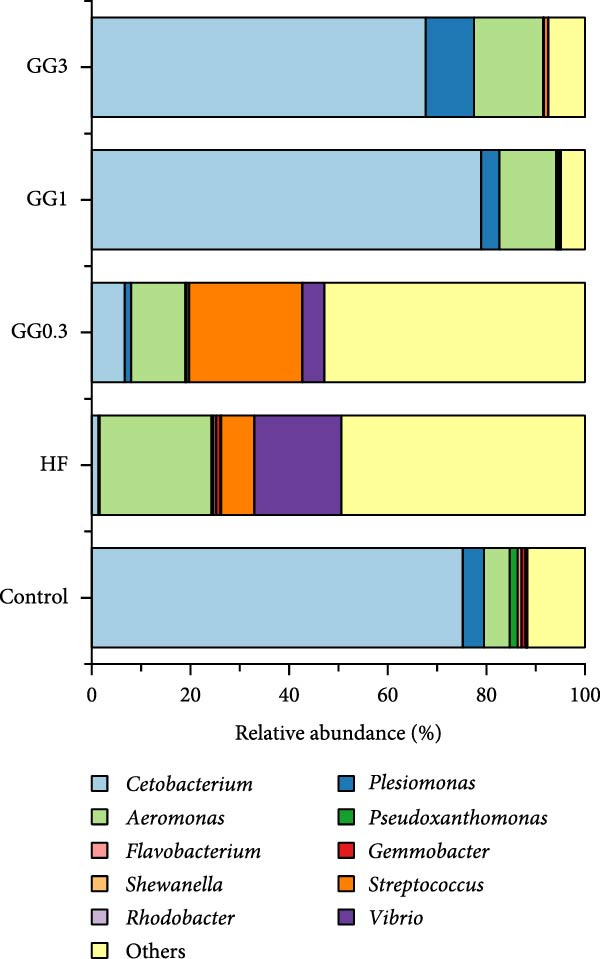
(C)

**Figure:**
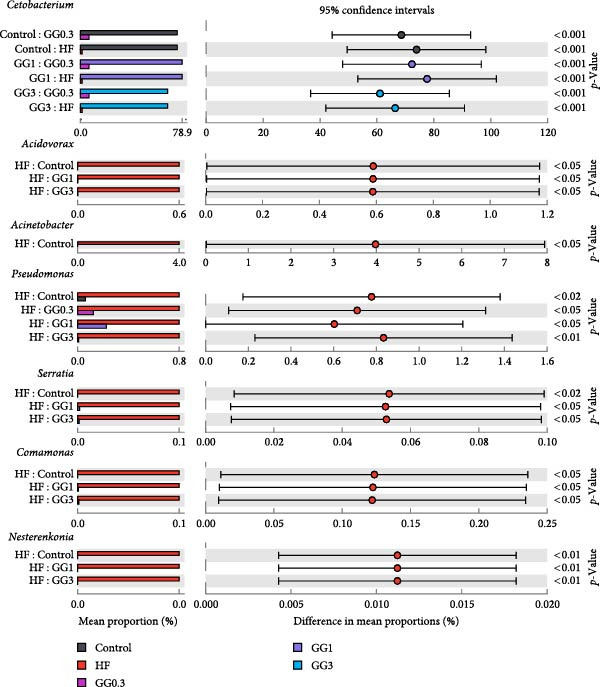
(D)

The microbial communities contained the following 10 most common genera: *Cetobacterium*, *Plesiomonas*, *Aeromonas*, *Pseudoxanthomonas*, *Flavobacterium*, *Gemmobacter*, *Shewanella*, *Streptococcus*, *Rhodobacter*, and *Vibrio* (Figure 2C). The HF diet significantly decreased *Cetobacterium* abundance as opposed to the control, but increased *Acidovorax*, *Acinetobacter*, *Pseudomonas*, *Serratia*, *Comamonas*, and *Nesterenkonia* abundance (*p* < 0.05). In fish given GG1 or GG3 diets, *Cetobacterium* abundance was higher than HF, but *Acidovorax*, *Acinetobacter*, *Pseudomonas*, *Serratia*, *Comamonas*, and *Nesterenkonia* abundance were lower (*p* < 0.05, Figure 2D).

### 3.3. Intestinal Histology

The HF diet induced a considerable decrease in villus height, villus width, and PR, and a rise in muscular thickness than control (*p* < 0.05, Figure 3). In contrast to the HF diet, the addition of 0.3% to 3% guar gum dramatically raised villus height and PR (*p* < 0.05). Moreover, the GG1 diet increased villus width (*p* < 0.05). Fish given the GG1 or GG3 diet exhibited a lower muscular thickness than those in the HF group (*p* < 0.05).

Figure 3Hindgut histology of common carp (100×, scale bar = 100 μm). (A) Control, (B) HF, (C) GG0.3, (D) GG1, (E) GG3, (F) villus height, (G) villus width, (H) muscular thickness, and (I) perimeter ratio. Bars with different lowercase letters exhibit significant differences (*p* < 0.05).

**Figure:**
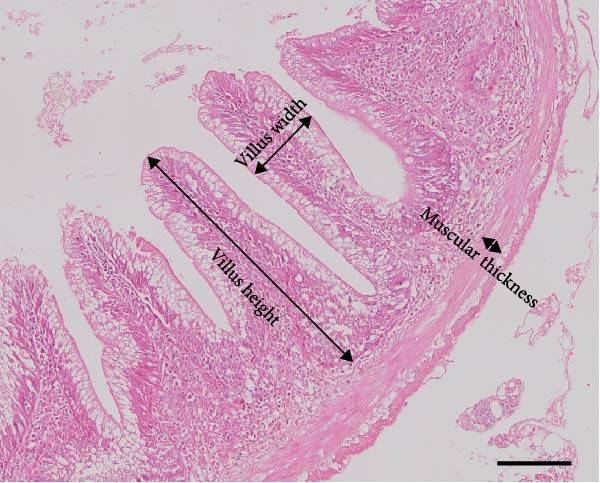
(A)

**Figure:**
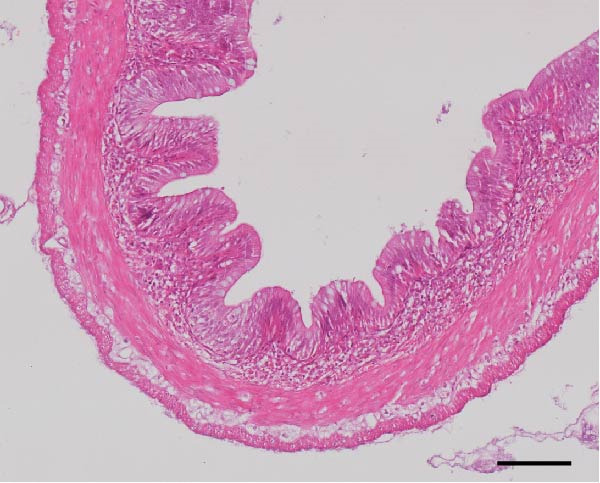
(B)

**Figure:**
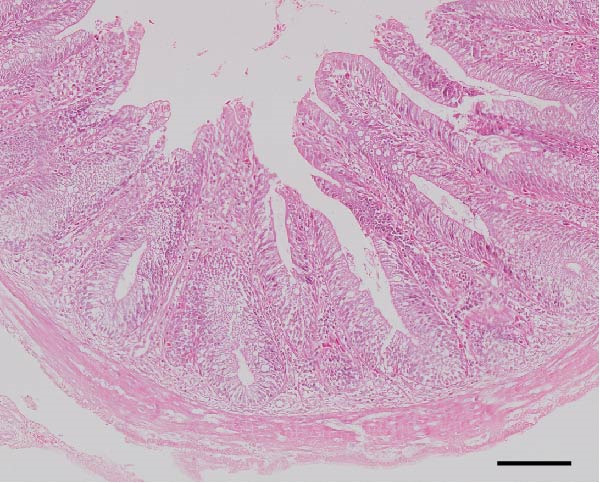
(C)

**Figure:**
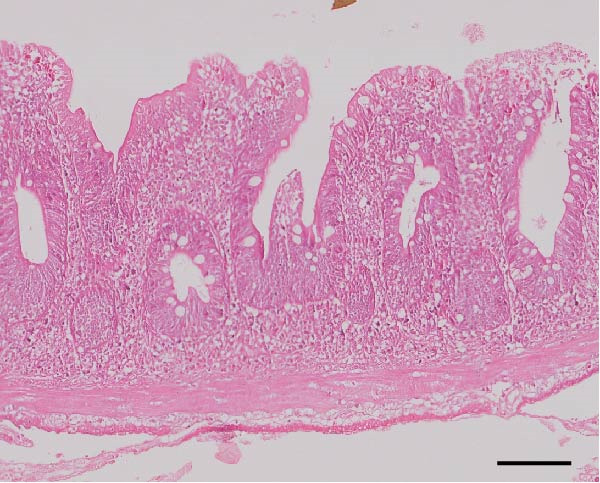
(D)

**Figure:**
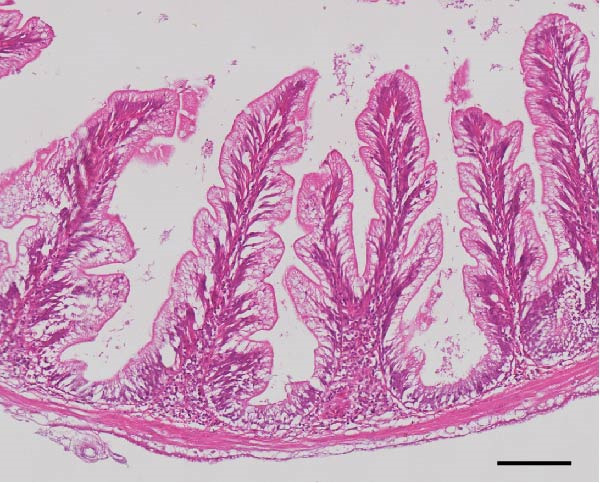
(E)

**Figure:**
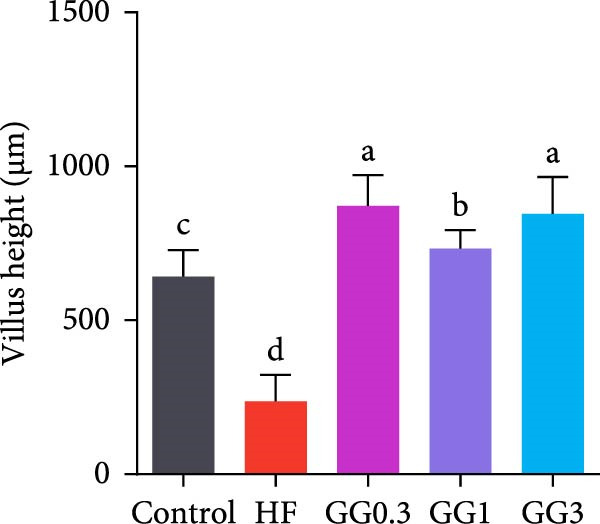
(F)

**Figure:**
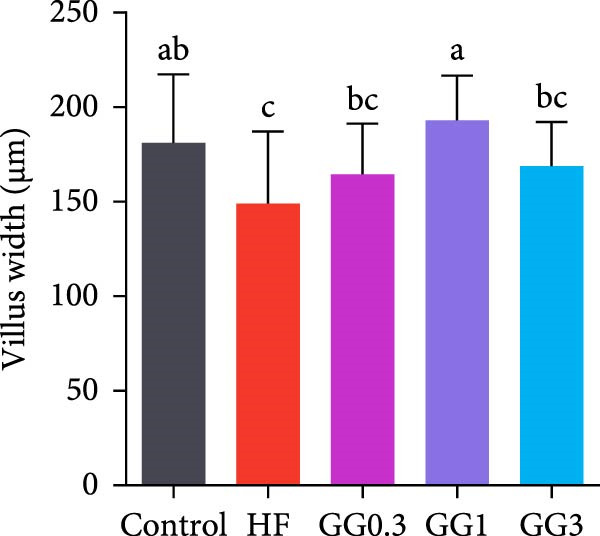
(G)

**Figure:**
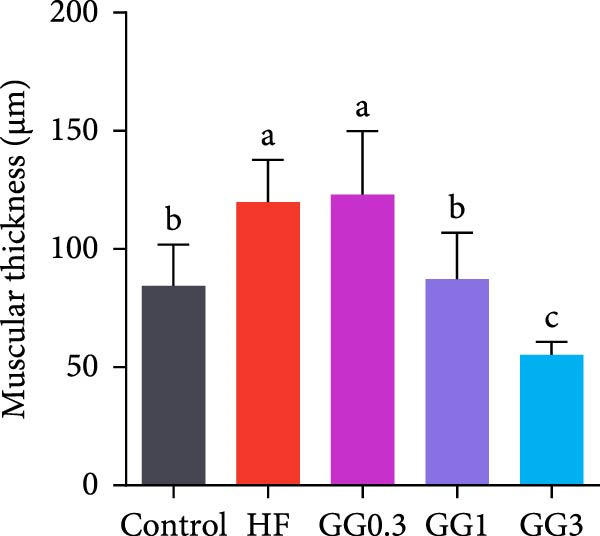
(H)

**Figure:**
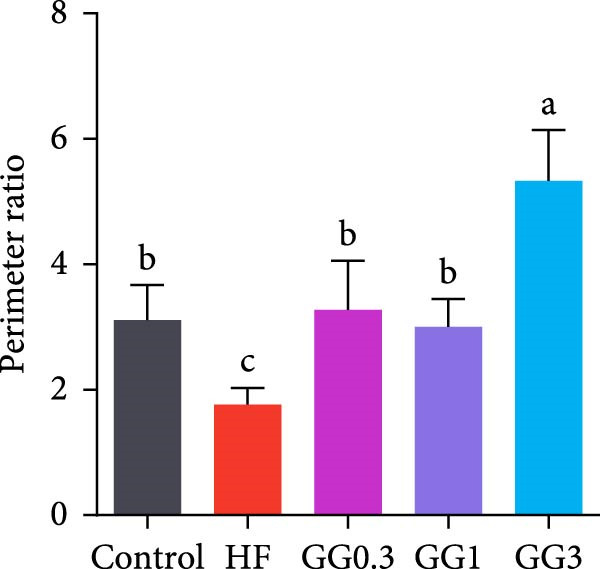
(I)

### 3.4. Expression of Gut Oxidative Stress and Barrier Function‐Related Genes

Fish fed HF exhibited a lower expression of *catalase (cat), nuclear factor erythroid* 2‐related factor 2 (*nrf2*), *zonula occludens-1* (*zo-1*), and occludin, and a higher expression of *kelch-like ECH-associated protein 1* (*keap1*), compared to the control group (*p* < 0.05, Figure 4). When compared to the HF diet, guar gum‐containing diets significantly elevated the expression of *nrf2* and *zo-1* and downregulated *keap1* expression (*p* < 0.05). In addition, fish fed the GG0.3 or GG1 diet had higher *cat* and *occludin* expression as opposed to those in the HF group (*p* < 0.05).

Figure 4Expression of genes linked to antioxidant enzymes and barrier function in the intestine. Bars with various lowercase letters show notable differences (*p* < 0.05). (A) *nrf2*, *nuclear factor erythroid* 2‐related factor 2; (B) *keap1*, *kelch-like ECH-associated protein 1*; (C) *cat*, *catalase*; (D) *occludin*; (E) *zo-1*, *zonula occludens-1*.

**Figure:**
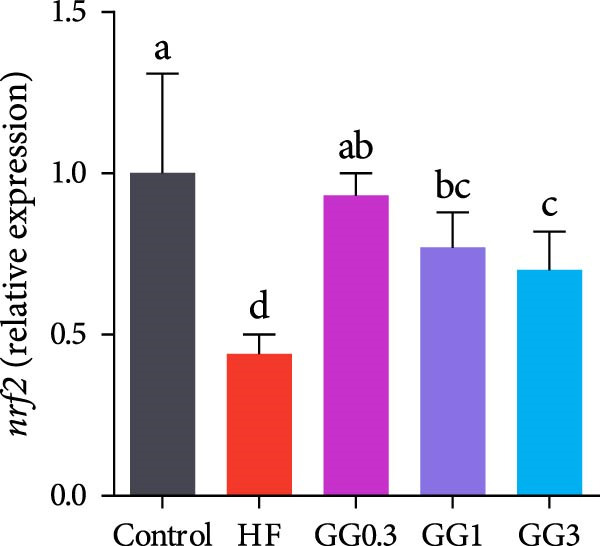
(A)

**Figure:**
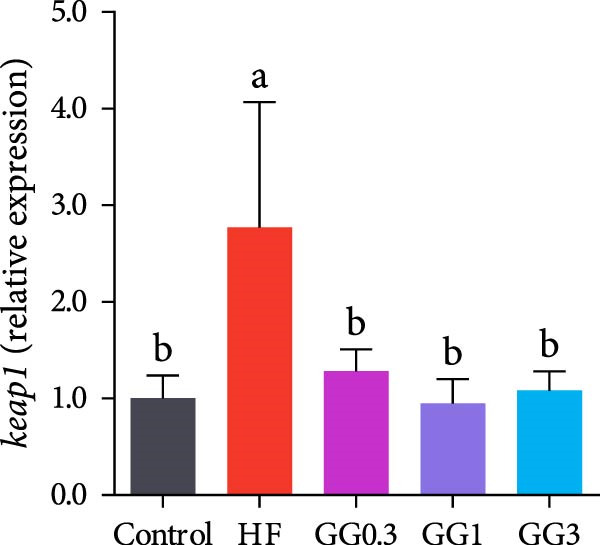
(B)

**Figure:**
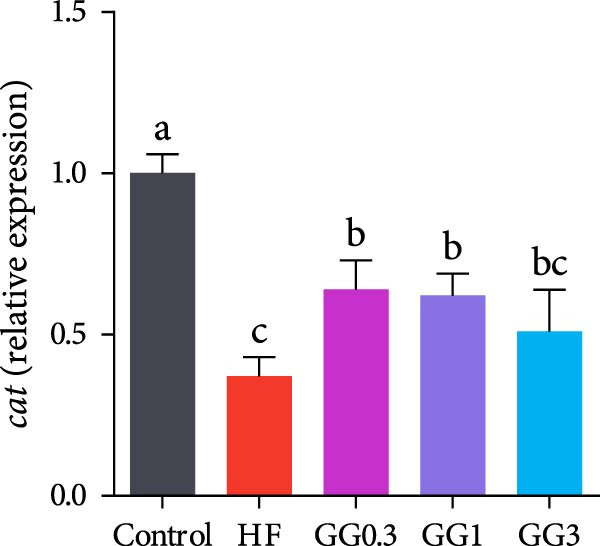
(C)

**Figure:**
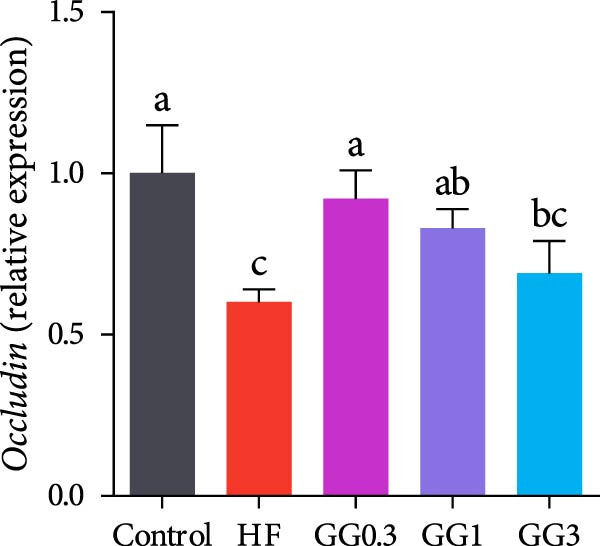
(D)

**Figure:**
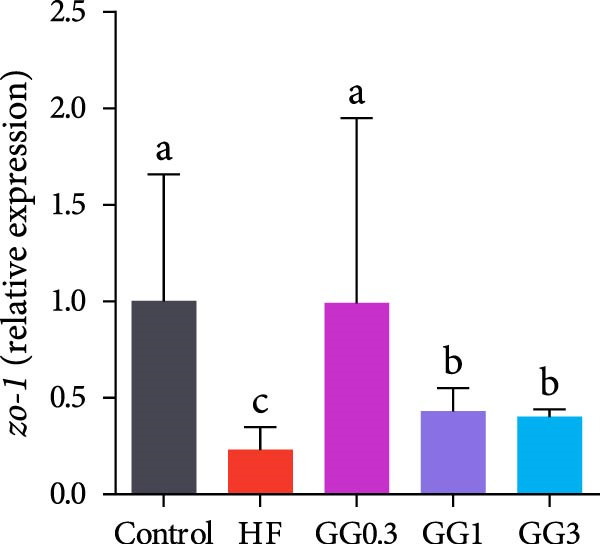
(E)

### 3.5. Expression of Intestinal Inflammation‐Related Genes

The expression of *interleukin-1β* (*il-1β*), *il-6*, *nuclear factor kappa B p65* (*nf-κb p65*), *il-8*, *myeloid differentiation factor 88* (*myd88*), *toll-like receptor 1* (*tlr1*), *tlr2*, and *tlr5* was elevated by the HF diet in contrast with the control (Figure 5, *p*  < 0.05). Dietary 0.3%–3% inclusion in HF diets dramatically reduced *tlr1*, *tlr5*, *myd88*, *il-1β*, *il-6*, and *il-8* expression (*p* < 0.05). Moreover, the GG0.3 and GG1 diets reduced *nf-κb p65* expression than HF (*p* < 0.05). Additionally, fish fed the GG1 diet showed lower *tlr2* expression than HF (Figure 5, *p*  < 0.05).

Figure 5Expression of genes linked to intestinal inflammation. Bars with different lowercase letters exhibit significant differences (*p* < 0.05). (A) *tlr1*, *toll-like receptor 1*; (B) *tlr2*, *toll-like receptor 2*; (C) *tlr5*, *toll-like receptor 5*; (D) *myd88*, *myeloid* differentiation factor 88; (E) *nf-κb* p65, *nuclear factor kappa B p65*; (F) *il-1β*, *interleukin-1β*; (G) *il-6*, *interleukin-6*; (H) *il-8*, *interleukin-8*; (I) *tnf-α*, *tumor necrosis factor α*.

**Figure:**
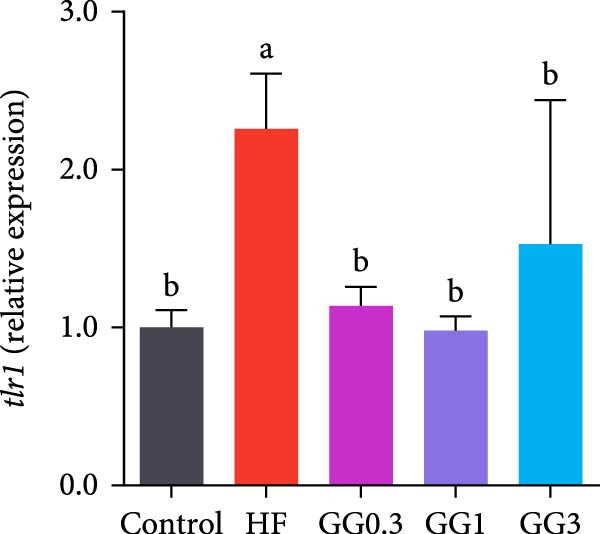
(A)

**Figure:**
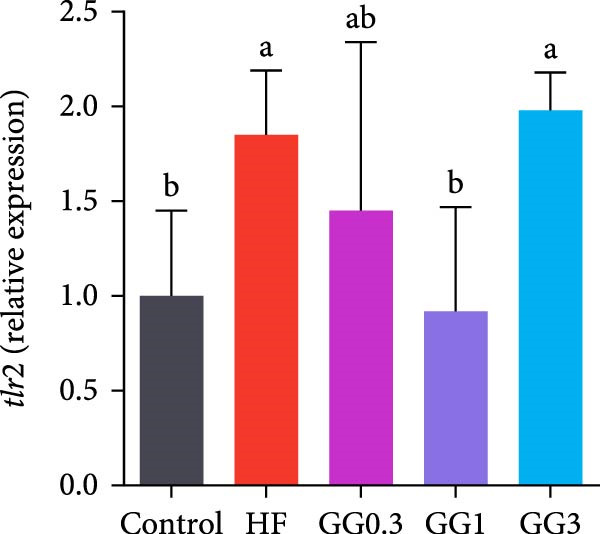
(B)

**Figure:**
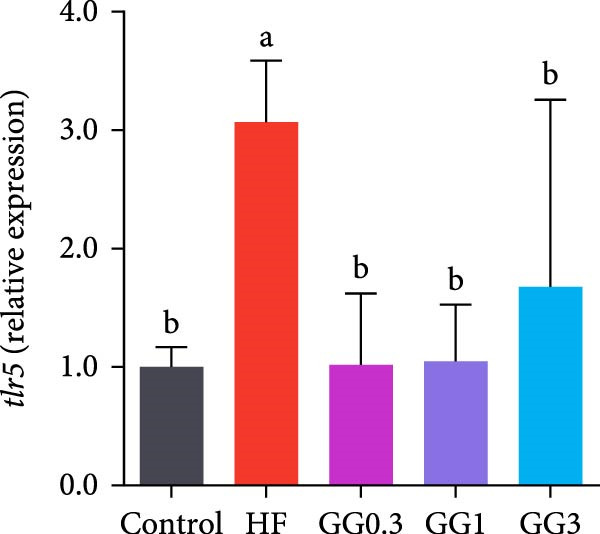
(C)

**Figure:**
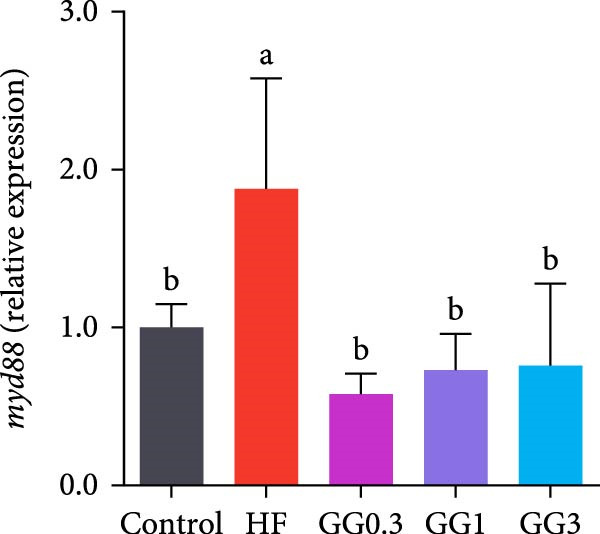
(D)

**Figure:**
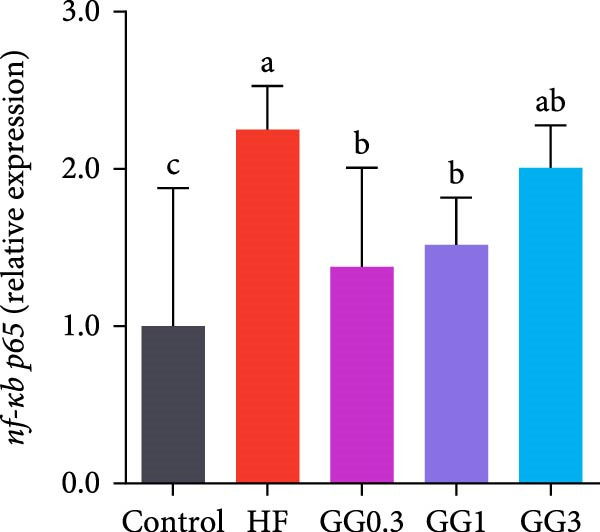
(E)

**Figure:**
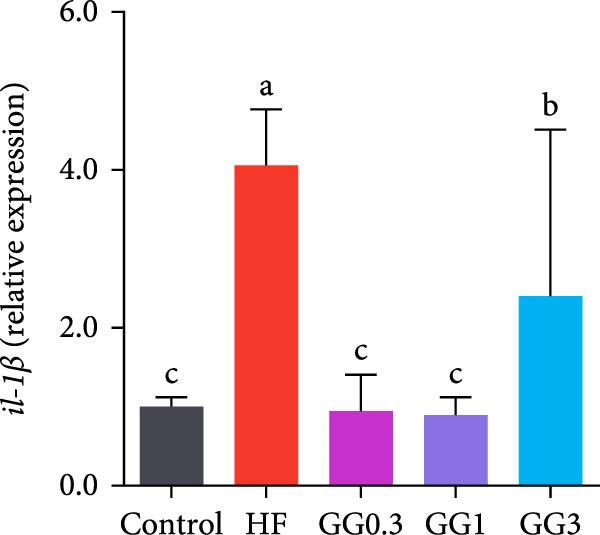
(F)

**Figure:**
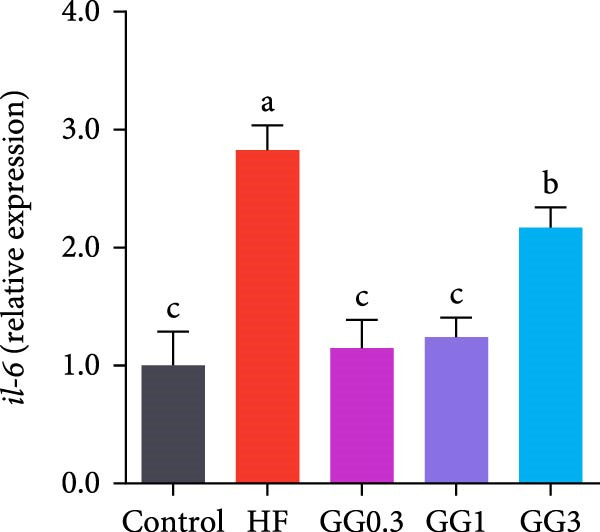
(G)

**Figure:**
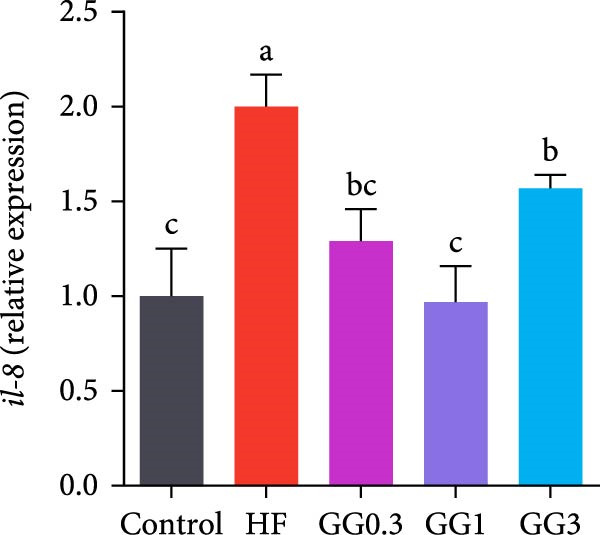
(H)

**Figure:**
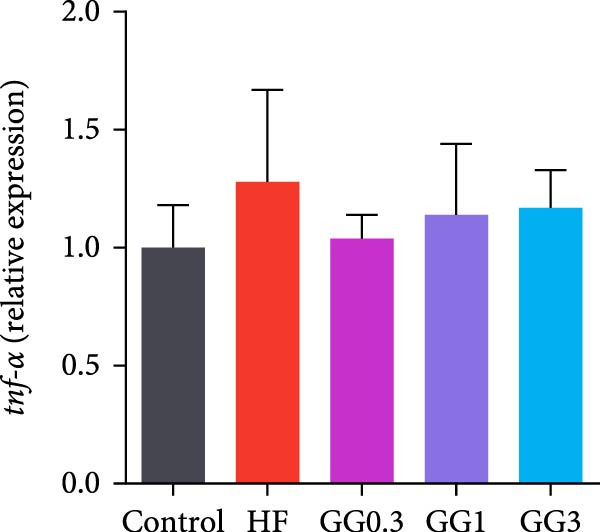
(I)

### 3.6. Plasma Antioxidant Capacity

Compared to the control, the HF diet significantly increased MDA and PC contents (*p* < 0.05). In contrast, guar gum diets elevated T‐SOD activity and reduced PC content relative to HF (*p* < 0.05). Furthermore, GG3‐fed fish showed higher CAT activity, while GG1‐fed fish exhibited lower MDA levels than HF (*p* < 0.05). Neither GSH concentrations nor GPx activity differed significantly between the treatments (Table 3).

**Table 3:** Antioxidant‐related parameters in the plasma of common carp.

Groups	Control	HF	GG0.3	GG1	GG3
T‐SOD (U/mL)	175.73 ± 10.09^ab^	129.8 ± 16.20^b^	191.57 ± 23.05^a^	206.27 ± 22.06^a^	196.4 ± 15.64^a^
CAT (U/mL)	4.68 ± 0.48^ab^	2.17 ± 0.40^b^	5.08 ± 1.89^ab^	5.15 ± 0.72^ab^	6.17 ± 1.23^a^
GSH (mg/L)	103.83 ± 5.65	136 ± 22.45	112.7 ± 5.07	118.93 ± 9.83	118.72 ± 9.45
GPx (U/mL)	973.45 ± 34.51	972.57 ± 49.63	944.07 ± 42.60	930.68 ± 32.17	920.18 ± 14.55
MDA (nmol/mL)	6.42 ± 0.65^ab^	8.21 ± 1.23^a^	6.83 ± 0.40^ab^	5.47 ± 0.58^b^	7.30 ± 0.65^ab^
PC (nmol/mg prot)	6.68 ± 2.79^b^	15.25 ± 1.23^a^	1.73 ± 0.53^b^	5.55 ± 4.40^b^	6.34 ± 1.85^b^

*Note:* Data are expressed as mean ± SD (*n* = 9). Values in the same row with different superscript letters differ significantly (*p* < 0.05).

Abbreviations: CAT, catalase; GPx, glutathione peroxidase; GSH, reduced glutathione; MDA, malonaldehyde; PC, protein carbonyls; T‐SOD, total superoxide dismutase.

### 3.7. Correlation Investigation of Tight Junction Proteins, Gut Microbiota, and Gut Inflammation

**Figure 6:**
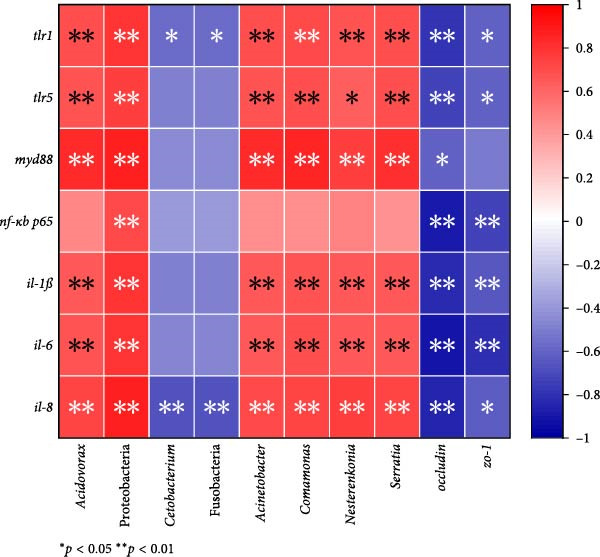
Correlation analysis between gut inflammation, tight junction proteins, and gut microbiota.

A Spearman’s correlation analysis showed that gut inflammation indicators (e.g., *tlr1*, *tlr5*, *nf-κb p65*, and *il-1β*) were positively correlated with occludin and zo‐1 and the abundance of Fusobacteria and *Cetobacterium* but negatively associated with the abundance of Proteobacteria, *Acdivorax*, *Acinetobacter*, *Comamonas*, and *Serratia* (*p* < 0.05, Figure 6). Based on the findings presented in this study, Figure S2 depicted a summary of guar gum’s beneficial effects on fish gut health.

## 4. Discussion

### 4.1. Adding Guar Gum to the Diet Reduced Oxidative Stress in Fish

Diets rich in lipids often induce oxidative stress in fish [21, 22]. The HF diet produced oxidative stress in this study, as evidenced by high plasma PC levels, a typical oxidative stress biomarker [23]. To counteract the stress, fish activate antioxidant defense systems, including SOD and CAT [24]. Compared to the HF diet, guar gum‐supplemented diets significantly reduced plasma PC contents and enhanced SOD activity. Furthermore, the GG1 or GG3 diet dramatically elevated *cat* expression in the gut relative to the HF group. These results demonstrate the ability of guar gum to mitigate oxidative stress, consistent with observations in largemouth bass (*Micropterus salmoides*) [25] and hamsters [26].

The antioxidant mechanism of guar gum involves the Nrf2‐Keap1 pathway. Nrf2 regulates cellular oxidant resistance and SOD and CAT expression while being negatively regulated by Keap1 [27]. In this study, the HF diet downregulated intestinal *nrf2* and increased *keap*1 expression in the intestine, consistent with observations in Nile tilapia (*Oreochromis niloticus*) [28] and abalone (*Haliotis discus hannai*) [29]. Nonetheless, guar gum diets dramatically suppressed *keap1* and activated *nrf2* in the gut, suggesting the antioxidant effect of guar gum via the Nrf2‐Keap1 pathway.

Guar gum consists of hydrophilic, nontoxic, and biodegradable polysaccharides [30]. Its fermentable nature allows gut microbes to break it and produce SCFAs, which are known to activate Nrf2 [31, 32]. Both research in mammals and fish has shown that fecal bacteria degrade guar gum primarily with acetate, followed by butyrate [33, 34]. Acetate and butyrate have been reported to promote *nrf2* expression in fish [35, 36]. Thus, guar gum may reduce oxidative stress by activating Nrf2 via its fermentation products.

### 4.2. Guar Gum Modulated Gut Microbiota Composition and Suppressed Inflammation

A balanced intestinal microbiota is critical for maintaining fish health. Consistent with the results in grass carp [5], HF diets increased Proteobacteria abundance and reduced Fusobacteria abundance in this study. A dysbiotic expansion of members of the phylum Proteobacteria has been proven to be closely connected with intestinal bowel disease [37, 38], while Fusobacteria improves aquatic animal health by contributing to vitamin and butyric acid synthesis [39]. However, dietary 1% or 3% guar gum supplementation in this research dramatically decreased Proteobacteria abundance and increased Fusobacteria abundance.

At the genus level, guar gum dramatically increased *Cetobacterium* abundance, which dominates the intestinal microbiota in many freshwater fish and is considered a beneficial microbe due to its ability to produce vitamin B12 [40]. Concurrently, guar gum decreased several potential pathogens including *Acidovorax*, *Acinetobacter*, *Pseudomonas*, *Serratia*, *Comamonas*, and *Nesterenkonia*. *Acidovorax* spp. can increase the risk of adenomas by causing local inflammation [41, 42]. *Acinetobacter* is a well‐known pathogen that often causes bacteremia and diarrhea [43]. *Pseudomonas* disrupts the intestinal epithelial barrier [44], while *Serratia* sp. causes severe infections in fish [45]. *Nesterenkonia* sp. has been linked to inflammatory disorders [46], and *Comamonas* sp. may lead to intestinal illness [47].

This microbiota remodeling directly correlates with reduced inflammation. In this study, the HF diet dramatically upregulated the expression of pro‐inflammatory cytokines (e.g., *il-8* and *il-1β*). However, supplementing HF with 0.3%–3% guar gum significantly lowered the expression of *tlr1*, *tlr5*, *myd88*, *il-1β*, *il-6*, and *il-8*. Moreover, administering 0.3%–1% guar gum within the HF markedly reduced *nf-κb p65* expression in the gut. These findings imply that the TLR‐Myd88‐NF‐*κ*B pathway is the mechanism by which guar gum can suppress inflammation.

The alleviated inflammation in fish receiving guar gum may be associated with the reduced abundance of Proteobacteria. The major, microbially associated molecular patterns on the proteobacterial cell surface are LPS [48] which are known to induce inflammation through the TLR‐Myd88‐NF‐*κ*B pathway [49, 50]. The correlation analysis in this work further confirmed a statistically significant positive relationship between Proteobacteria abundance and inflammation.

### 4.3. Guar Gum Alleviated HF Diet‐Induced Damage to Gut Morphology and Barrier Function

The gut is a versatile organ that performs digestion, absorption, and barrier function [51]. In this investigation, the HF diet decreased the villus height, villus width, and PR. Increases in villus height and width can improve fish absorption and utilization of nutrients [52]. A high PR always implies a high absorptive surface area [53]. The villus height and PR increased in fish fed a HF diet supplemented with 0.3%–3% guar gum, suggesting that guar gum enhanced nutrient absorption and digestion. Our findings are consistent with those from investigations of grass carp (*Ctenopharyngodon idella*) [5] and largemouth bass [54].

Tight junctions are necessary for the intestinal barrier to function and serve as a physical barrier to luminal inflammatory chemicals [55]. Occludin and ZO‐1 are critical tight junction proteins essential for intestinal barrier integrity and function [56]. The HF diet dramatically reduced *zo-1* and *occludin* expression in this investigation, indicating that it damaged the physical barrier function. Comparable outcomes have also been reported for grass carp [5] and blunt snout bream (*Megalobrama amblycephala*) [57]. Nevertheless, *occludin* and *zo-1* expression was markedly elevated by 0.3%–1% guar gum, suggesting that guar gum improved the intestinal barrier. Correlation analysis in this work revealed that *occludin* and *zo-1* were critical in counteracting inflammation.

Guar gum’s chemical and physical features may account for its favorable benefits on gut morphology, mucosal barrier, and gut inflammation. Guar gum’s viscosity not only decreases gut transit time but also creates a more favorable environment for nutrient absorption, triggering an adaptive increase in villus height and PR to maximize nutrient absorption [58]. Furthermore, fermentation products of guar gum (e.g., acetate and butyrate) are key energy sources for epithelial cells and may provide energy while also stimulating intestinal epithelial cell proliferation and differentiation, resulting in enhanced villus height and PR [59]. Acetate and butyrate have been reported to increase villus height in fish [60, 61]. These same fermentation products can be ascribed to the overexpression of tight junction proteins and the downregulation of proinflammatory cytokines genes, as they have been shown to boost occludin and zo‐1 expression while decreasing proinflammatory cytokine expression in fish [61–63].

## 5. Conclusion

Supplementation of HF diets with 0.3%–1% guar gum decreased oxidative stress through Nrf2‐Keap1 pathway activation, enhanced mucosal barrier function by upregulating tight junction proteins, and reduced gut inflammation by inhibiting the TLR‐Myd88‐NF‐*κ*B pathway. Furthermore, administering 1% to 3% guar gum in HF diets improved gut dysbiosis. These results underline the protective role of guar gum against HF feed‐induced intestinal injury in common carp, demonstrating that its benefits extend beyond dietary fiber to include significant modulation of gut microbiota and enhancement of gut morphology. This multifaceted role of guar gum is crucial for promoting overall gut health and offers potential applications in aquaculture nutrition and management.

## Conflicts of Interest

The authors declare no conflicts of interest.

## Author Contributions

**Weijun Chen**: writing ‐ original draft, funding acquisition. **Shiyang Gao**: methodology, investigation, visualization, formal analysis. **Xiaoyu Zhao**: investigation, formal analysis, writing ‐ original draft.; **Na Zhao**: writing ‐ review and editing, conceptualization. **Ping Sun**: resources, data curation.; **Lei Han**: visualization, resources.

## Funding

This study was funded by the National Natural Science Foundation of China, Grant 32202952.

## Supporting Information

Additional supporting information can be found online in the Supporting Information section.

## Supporting information


**Supporting Information** Table S1. Chemical and physical properties of the guar gum extracted from the Cyamopsis tetragonolobus seeds. Table S2. Primer sequences for RT‐PCR in the experiment. Figure S1. Gut beta diversity in common carp. (A) UPGMA clustering tree based on Jaccard coefficient index. (B) Principal coordinate analysis (PCoA) based on weighted Unifrac distance. Figure S2. A summary of guar gum’s beneficial effects on fish gut health.

## Data Availability

Data are available upon reasonable request.
