# Supplementary material for: Adding Guar Gum to High‐Fat Diets Ameliorates Fish Growth, Gut Histology, Gut Microbiota Composition, and Intestinal Inflammation in Common Carp
Source: Aquac Nutr. 2026 Jan 16;2026:2722361. doi: 10.1155/anu/2722361 (PMC12811151; doi:10.1155/anu/2722361)
Supplement: Supplementary file 1 — Supporting Information Table S1. Chemical and physical properties of the guar gum extracted from the Cyamopsis tetragonolobus seeds. Table S2. Primer sequences for RT‐PCR in the experiment. Figure S1. Gut beta diversity in common carp. (A) UPGMA clustering tree based on Jaccard coefficient index. (B) Principal coordinate analysis (PCoA) based on weighted Unifrac distance. Figure S2. A summary of guar gum’s beneficial effects on fish gut health. [file ANU-2026-2722361-s001.docx]

**Supplementary Table S1** Chemical and physical properties of the guar gum extracted from the *Cyamopsis tetragonolobus* seeds

| Parameter | Content |
| --- | --- |
| Moisture (%) | 10.0 |
| Crude protein (% dry weight) | 5.9 |
| Crude lipid (% dry weight) | 0.9 |
| Ash (% dry weight) | 0.8 |
| Galactomannan content (% dry weight) | 88.5 |
| Mannose/galactose ratio | 1.6:1 |
| pH | 6.4 |
| Viscosity (mPa·s) | 5200 |

**Supplementary Table S2** Primer sequences for RT-PCR in the experiment

| Gene | Sequence (5’-3’) | GenBank ID |
| --- | --- | --- |
| *cat* | F: TTCCTGTGGGACGCCTTGT | JF 411604.1 |
|  | R: TCCGAGCCGATGCCTATGT |  |
| *keap1* | F: CAGTGGGCGAGAAGTGT | JX 470752.1 |
|  | R: TTTGATGGCTCCAGGTT |  |
| *myd88* | F: CGCCGAAATGATGGACTTCAC | HQ380208.1 |
|  | R: TCTACTGTTGCCTCTGGACG |  |
| *nf-κb p65* | F: AGAAGAGCAACGACACCACAA | MN167531.1 |
|  | R: TTGTACGGCTGGTTCTTGGTT |  |
| *il-1β* | F: AAGGAGGCCAGTGGCTCTGT | AB010701 |
|  | R: CCTGAAGAAGAGGAGGCTGTCA |  |
| *il-6* | F: CATCTGGGGACGAGGTTCAG | XM_019073058.1 |
|  | R: AGGGTTTGAGGAGAGGGGTT |  |
| *il-8* | F: GTCTTAGAGGACTGGGTGTA | EU011243.1 |
|  | R: ACAGTGTGAGCTTGGAGGGA |  |
| *nrf2* | F: TTCCCGCTGGTTTACCTTAC | JX462955 |
|  | R: CGTTTCTTCTGCTTGTCTTT |  |
| *occludin* | F: GACGCCATGGATGAGTACAA | KF975606.1 |
|  | R: GTGGTTGAGTTTGGCTTTCAG |  |
| *tnf-α* | F: AGCCAGGTGTCTTTCCACAT | XM_019088899.1 |
|  | R: ATGTAGCCGCCATAGGAATCG |  |
| *tlr1* | F: TGGCCCCAGGATTACACTTG | XM_019083628.1 |
|  | R: CCAGAGCAGTTCCTACCACAA |  |
| *tlr2* | F: TGTGCGACACTCCATTCACT | HQ731681.1 |
|  | R: TCCATGCTTTGGTCATGTGC |  |
| *tlr5* | F: GAAGTAGTGAAAAGCACCTCGG | LC150765.1 |
|  | R: GATTTACATGCGTGGGCACT |  |
| *zo-1* | F: GATATGTTCGGAGGTGCGCT | KY290394.1 |
|  | R: ATGTTGCATGGTGCTTGCTG |  |
| *β-actin* | F: TTGCTCCCTCCACCATGAAG | JQ619774.1 |
|  | R: ACTCCTGCTTGCTGATCCAC |  |

Notes: *cat*, catalase; *keap1*, Kelch-like ECH-associated protein 1; *myd88*, myeloid differentiation factor 88; *nf-κb p65*, nuclear factor kappa B; *il-1β*, interleukin-1β; *il-6*, interleukin-6; *il-8*, interleukin-8; *nrf2*, nuclear factor erythroid 2-related factor 2; *tnf-α*, tumor necrosis factor α; *tlr1*, toll-like receptor 1; *tlr2*, toll-like receptor 2; *tlr5*, toll-like receptor 5; *zo-1*, zonula occludens-1.

**Supplementary Figure S1**


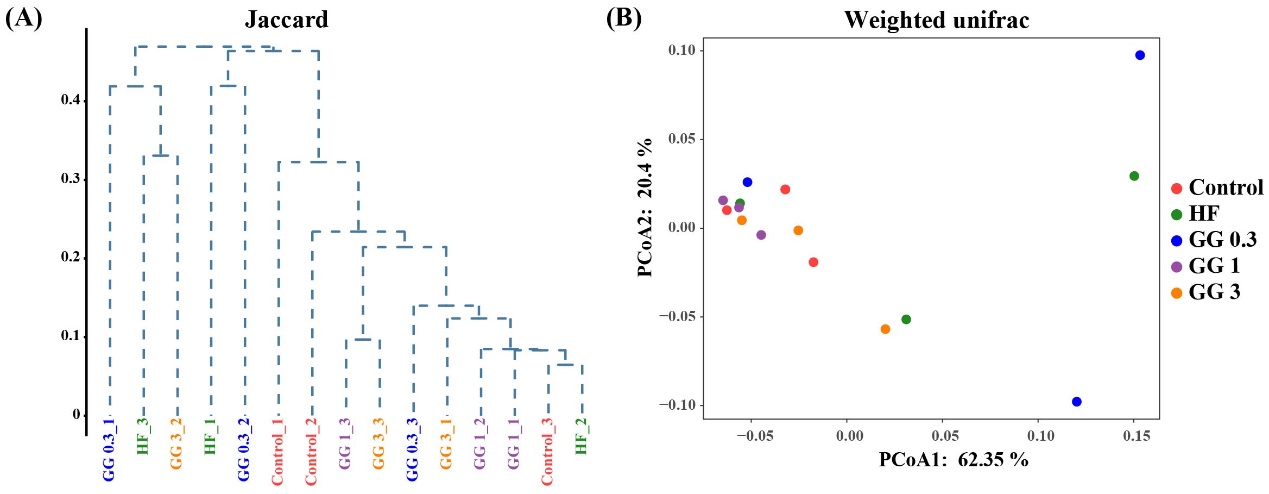


Fig. S1 Gut beta diversity in common carp. (A) UPGMA clustering tree based on Jaccard coefficient index. (B) Principal coordinate analysis (PCoA) based on weighted Unifrac distance.

**Supplementary Figure S2**


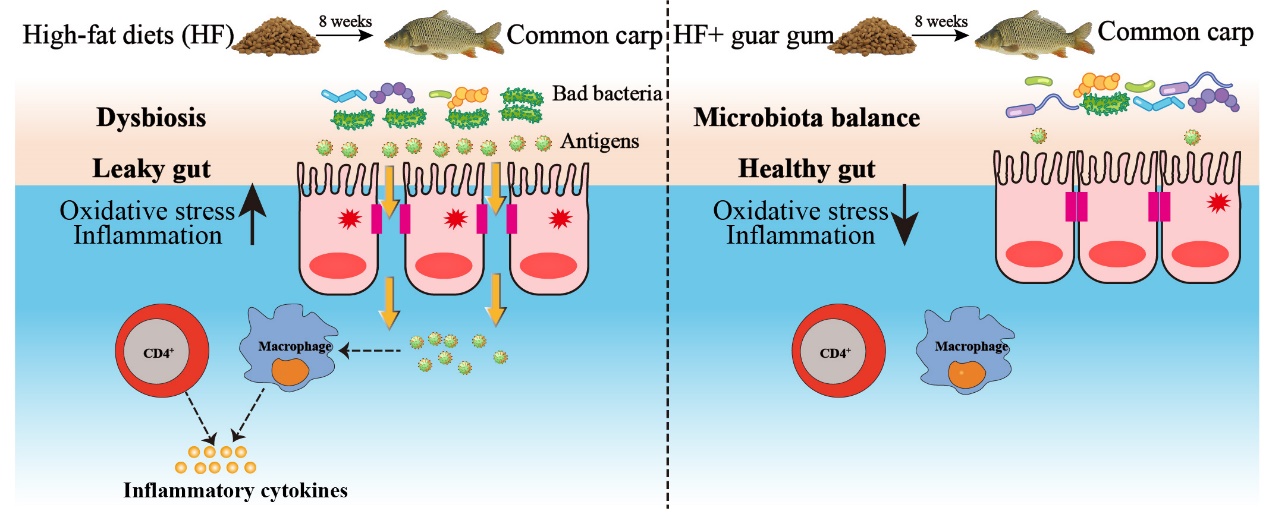


Fig. S2 A summary of guar gum’s beneficial effects on fish gut health.
